# The prognostic value of clinical frailty scale and outcomes in older patients undergoing left ventricular assist device implantation

**DOI:** 10.1002/agm2.12227

**Published:** 2022-11-17

**Authors:** Temitope Ajibawo, Priyank Chauhan, Radha S. Gopalan, Nimit K. Agarwal

**Affiliations:** ^1^ Division of Geriatric Medicine, Department of Internal Medicine Banner University Medical Center Phoenix Arizona USA; ^2^ Division of Cardiology, Department of Internal Medicine Banner University Medical Center Phoenix Arizona USA

**Keywords:** clinical frailty scale, left ventricular assist device, mortality, outcomes

## Abstract

**Objectives:**

Heart failure impacts patients’ functional capabilities, ultimately leading to frailty. The use of a left ventricular assist device (LVAD) is acceptable as both destination therapy and bridge to transplant in heart failure management. We aim to evaluate the prognostic value of the Clinical Frailty Scale (CFS) on outcomes in older patients undergoing implantation of LVAD.

**Methods:**

We conducted a retrospective chart review of patients ≥ 60 years old that underwent LVAD implantation at our medical center from May 1, 2018, to October 30, 2020. CFS was retrospectively assigned before LVAD placement and CFS scores > 4 was considered frail. Kaplan–Meier curves and Cox regression were used to analyze 1‐year survival estimates.

**Results:**

Forty percent of the cohort was classified as frail according to CFS. Thirty‐day re‐admission rates were comparable between frail and non‐frail patients (46% vs 35%; *P* = 0.419). 1‐year survival was lower in the frail vs non‐frail group (log rank, *P* = 0.017). On Cox analysis, only frailty was associated with 1‐year post‐intervention mortality (hazard ratio [*HR*] = 5.64, 95% confidence interval [CI] = 1.131–28.212; *P* = 0.035).

**Conclusions:**

CFS‐defined frailty was associated with increased risk of 1‐year mortality after LVAD implantation. CFS may be a valuable tool in the frailty assessment for risk stratification of patients undergoing LVAD implantation. Multicenter studies are required to validate these findings.

## Graphical abstract

The main finding in our study is that Clinical Frailty Scale‐defined frailty status was predictive of 1‐year post‐interventional mortality after left ventricular assist device implantation.

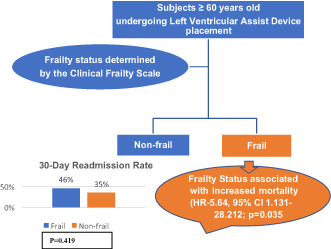

## 1 INTRODUCTION

Left ventricular assist device (LVAD) implantation has been reported to be associated with improved functional capability, survival, and quality of life in subjects with stage D heart failure.^1^, ^2^, ^3^ In addition, LVAD as destination therapy (DT) is acceptable for the management of patients with advanced heart failures that are not eligible for heart transplantation.^4^, ^5^ Although, not all patients are alike; physiological determinants, including frailty, should be considered in the risk stratification of patients with advanced heart failure to determine those likely to benefit from such interventions.^4^

Frailty is a medical syndrome of decreased resistance to stressors, portending vulnerability to adverse outcomes after medical or surgical stressful conditions.^6^ Frailty is prevalent in patients with chronic heart failure and is linked with a higher risk of mortality and heart failure hospitalizations.^7^, ^8^, ^9^ This study aims to investigate the prognostic significance of the clinical frailty scale (CFS) on short‐ and long‐term outcomes after LVAD implantation.

## 2 METHODS

We conducted a retrospective chart review of prospectively collected data of subjects ≥ 60 years old who underwent LVAD implantation at our academic medical center between May 1, 2018, and October 30, 2020. The LVAD types that were implanted include Heart Mate (HM) III and Heartware Ventricular Assist Device (HVAD). The data were collected, stored, and managed in a secured database, REDCap.^10^, ^11^ Our study received approval from the Institutional Review Board of the Banner University Medical Center, Phoenix, Arizona.

### 2.1 Frailty assessment

**FIGURE 1:**
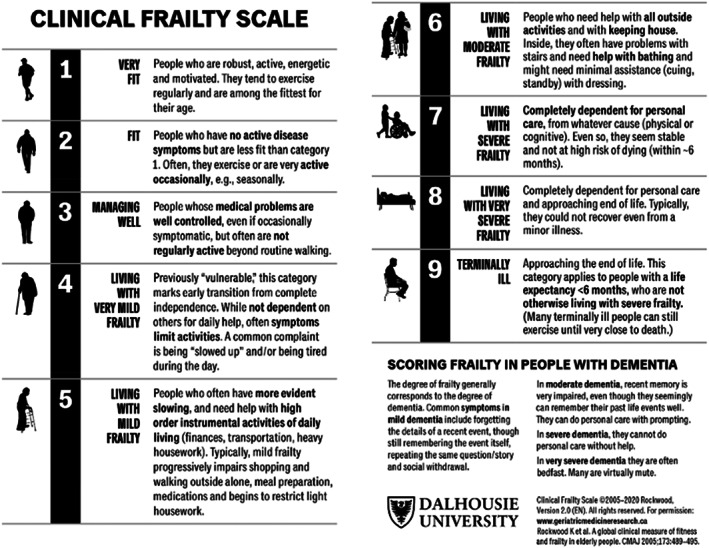
Clinical Frailty Scale chart. This is the assessment chart used for our frailty assessment

Frailty was evaluated using the CFS, which originated from the Canadian Study of Health and Aging.^12^ The CFS is a clinical‐based 9‐point scale with scores between 1 (very fit) and 9 (terminally ill) according to their level of dependence, functional capacity, comorbidities, and cognitive status, as shown in Figure 1.^13^ CFS is an easy‐to‐perform, partially quantitative, clinical judgment tool that evaluates frailty based on the patients’ mobility, activity, and independence level during daily physical activity.^14^ CFS does not require physical testing and can be completed within a minute, making it very appealing for clinical practice.^15^ In addition, it does not require any assessment of muscle strength, mobility, or nutritional status.^14^ CFS possesses good inter‐rater reliability and has been validated for frailty assessment in hospitalized older patients.^16^, ^17^

Two geriatric medicine fellows skilled in frailty assessment reviewed the existing medical records of physical activity, level of independence to do basic activities of daily living (ADLs) or instrumental activities of daily (IADLs), fitness level, and cognitive status documentation by physical therapists, occupational therapists, physicians, and nurses to assign CFS scores. The frailty assessment was based on the information from a 2–3‐week period before the index hospitalization for the LVAD placement. A senior geriatrician adept in frailty assessment supervised the process of CFS evaluation. The geriatric fellows were unaware of the outcomes of LVAD implantation to avoid introducing bias. We used the CFS application on iOS to calculate the scores based on the clinical information obtained from the chart. First, we scored each patient from CFS 1 to 9, as showed in Figure 1. Then, because of the small sample size, we categorized each subject into non‐frail (CFS = 1–4) and frail (CFS = 5–9). In addition, we envisaged that there might not be enough events for each CFS score during analysis.

The baseline characteristics and postoperative outcomes were obtained from patient's medical records by the heart failure team. The baseline characteristics include age, gender, device type, type of cardiomyopathy, serum albumin level, serum prealbumin level, total serum protein level, Interagency Registry for Mechanically Assisted Circulatory Support (INTERMACS) scores at the time of LVAD implantation, and pre‐operative chronic kidney disease (CKD). We defined pre‐operative chronic renal failure as Chronic Kidney Disease Epidemiology Collaboration (CKD‐EPI) < 60 ml/min/1.73 m^2^. Other comorbidities were obtained from clinician's diagnoses in the electronic chart. The left ventricular ejection fraction (%) represents the baseline left ventricular ejection fraction recorded in the chart determined by echocardiography interpreted by the cardiologists. The postoperative variables are the time to extubation (days), 6‐month survival, 12‐month survival, postoperative length of stay, discharge status, and 30‐day re‐admission. The time to extubation (days) was defined as “time from intubation to time to extubation following LVAD placement” and postoperative length of stay was defined as “time from date of LVAD implantation” to discharge day from the hospital following LVAD placement (in terms of days). The 30‐day re‐admission represents rehospitalization for any purpose between 0 and 30 days after discharge for index hospitalization of LVAD placement.

### 2.2 Statistical analysis

Baseline characteristics were presented for categorical variables as frequencies (percentage) and continuous variables as median (interquartile range). We used the Wilcoxon rank‐sum test to compare non‐normally distributed continuous variables between the two groups‐frail vs non‐frail. We used Fisher's exact test for non‐normally distributed data.

Kaplan Meier curves were generated to obtain survival estimates, and the log‐rank test was used to compare survival estimates between the frail and non‐frail groups. We defined survival time as the time from the date of LVAD implantation and the date of death or day of censoring (365 days after LVAD placement). Next, Cox regression model was used to study the relationship between mortality and covariates. A *P* value of ≤ 0.05 was regarded as statistically significant. Analyses were conducted with IBM SPSS version 28 (Armonk, NY).

## 3 RESULTS

Thirty patients ≥ 60 years old underwent LVAD implantation during the study period, 24 (80%) were men, 65% of the LVAD implanted were HM III devices. Of the 30 patients, 12 (40%) were assessed to be frail at baseline. Frailty was associated with HVAD, and higher serum total protein as shown in Table 1.

**TABLE 1:** Baseline pre‐operative variables between frail and non‐frail patients according to the Clinical Frailty Scale

Pre‐operative variables	Overall, n = 30	Non‐frail, n = 18	Frail, n = 12	*P* value
Device type				
HM III	19 (63)	15 (83)	4 (33)	0.009
HVAD	11 (37)	3 (17)	8 (67)	
Age, y	70.5 (66.0, 74.0)	70.5 (66.0, 74.0)	70.5 (66.2, 71.8)	0.700
Gender				
Male	24 (80)	14 (78)	10 (83)	1.000
Female	6 (20)	4 (22)	2 (17)	
Type of cardiomyopathy				
Ischemic etiology	20 (67)	11 (61)	9(75)	0.800
Laboratory parameters				
Serum albumin (g/dl)	3.6 (3.1, 3.9)	3.7 (3.0, 4.1)	3.6 (3.5, 3.7)	0.800
Hypoalbuminemia	10 (34)	8 (47)	2 (17)	0.130
Serum prealbumin (mg/dl)	15.0 (13.0, 20.0)	15.0 (13.0, 20.0)	15.5 (14.2, 18.5)	1.000
Total serum protein (g/dl)	6.3 (5.8, 6.9)	6.0 (5.8, 6.7)	6.6 (6.0, 7.2)	0.031
Comorbidities				
DM	13 (43)	7 (39)	6 (50)	0.547
COPD	8 (27)	5 (28)	3 (25)	1.000
Malignancy	5 (17)	4 (22)	1 (8)	0.622
Depression	4 (13)	2 (11)	2 (17)	1.000
CVA	1 (3)	0 (0)	1 (8)	0.400
Atrial fibrillation	17 (57)	8 (44)	9 (75)	0.098
CKD	8 (27)	5 (28)	3 (25)	1.000
INTERMACS score	3.0 (3.0, 3.0)	3.0 (2.7, 3.0)	3.0 (3.0, 3.0)	0.800
LVEF (%)	15 (10, 20)	15 (10, 20)	15 (10, 24)	0.950

*Note*: Median (interquartile range [IQR]) for continuous variables; n (%) for categorical variables.

Abbreviations: CKD, chronic kidney disease; COPD, chronic obstructive pulmonary disease; CVA, cerebrovascular accident; DM, diabetes mellitus; HM III, Heart Mate III; HVAD, Heart Ware Ventricular Assist Device; INTERMACS, Interagency Registry for Mechanically Assisted Circulatory Support; LVEF, left ventricular ejection fraction.

### 3.1 Frailty and postoperative outcomes

Postoperative outcomes in terms of postoperative length of stay (LOS; days) and time to extubation (days) did not differ significantly between the two groups in Table 2. However, at 12 months, frail patients had a statistically significant lower survival rate. Other results are shown in Table 2.

**TABLE 2:** Postoperative outcomes between frail and non‐frail patients according to the Clinical Frailty Scale

Postoperative variables	Overall (n = 30)	Non‐frail (n = 18)	Frail (n = 12)	*P* value
Time to extubation, days	1.0 (1.0, 2.0)	1.0 (1.0, 2.0)	1.0 (1.0, 2.0)	0.707
Postoperative length of stay, days	19.5 (15.2, 32.0)	19.0 (15.0, 33.0)	20.0 (16.0, 29.0)	0.893
6‐mo survival	26 (87)	17 (94)	9 (75)	0.274
12‐mo survival	22 (73)	16 (89)	6 (50)	0.034
Discharge to				
Home	8 (27)	8 (44)	0 (0)	0.009
Rehabilitation facility	18 (60)	9 (50)	9 (75)	
Died in the hospital	4 (13)	1 (6)	3 (25)	
30‐day re‐admission	11 (42)	6 (35)	5 (46)	0.419

*Note*: Median (interquartile range [IQR]) for continuous variables; n (%) for categorical variables.

**FIGURE 2:**
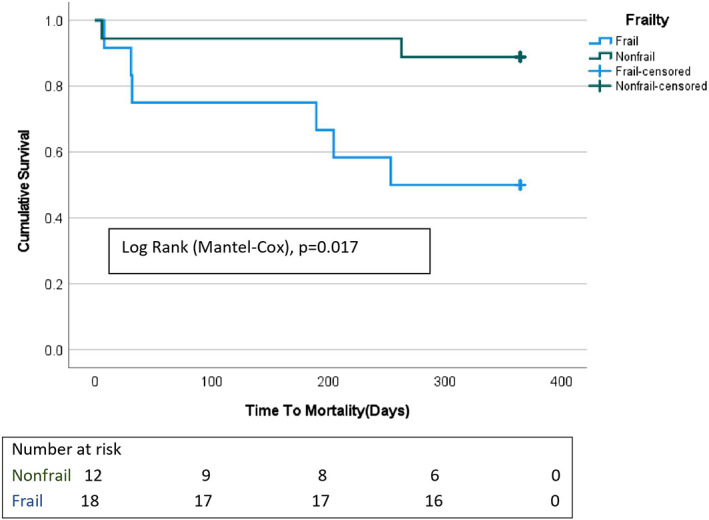
Kaplan–Meier Survival estimates. This figure shows the 365‐day post‐LVAD placement cumulative survival in frail vs non‐frail patients. LVAD, left ventricular assist device.

We observed a statistically significant difference in 1‐year survival between the frail and non‐frail groups (Log‐rank test (Mantel‐Cox), *P* = 0.017), as shown in Figure 2 below. We also studied the impact of frailty status (frail vs non‐frail), age (≤ 70 years vs > 70 years), gender (male vs female), hypoalbuminemia (no vs yes), INTERMACS profile score, device type (HM III vs HVAD), total serum protein level (< 6 vs ≥ 6 g/dl), type of cardiomyopathy (non‐ischemic vs ischemic), pre‐operative CKD (yes vs no), and prealbumin (< 18 vs ≥ 18 mg/dl), and time to extubation (days) on post‐intervention mortality using Cox regression. Frailty was the only variable associated with increased post‐LVAD intervention mortality (hazard ratio [*HR*] = 5.64, 95% confidence interval [CI] = 1.131–28.212; *P* = 0.035).

## 4 DISCUSSION

Our analysis showed statistically significant association between CFS‐defined frailty and 1‐year post‐intervention mortality (*HR* = 5.64, 95% CI = 1.131–28.212; *P* = 0.035). These findings are consistent with previous studies that have reported significant associations between frailty and long‐term post‐LVAD mortality.^18^, ^19^, ^20^, ^21^ For example, one of the studies by Dunlay et al, used the Frailty Index based on 31 deficits derived from the medical chart to evaluate frailty and showed a greater than three times higher risk of mortality in the highest frailty tertile.^18^ In another study, the investigators used handgrip strength, a marker of frailty, and reported an increased risk of mortality with handgrip strength < 25% of body weight following ventricular assist device (VAD) implantation.^19^ Few studies have also reported nonsignificant results for the association of frailty with long‐term mortality post‐LVAD intervention.^22^, ^23^, ^24^

The increased mortality risk described in our study may be due to multiple causes. First, frail patients often take longer to recuperate after insults from surgeries. Therefore, they are vulnerable to postoperative adverse events resulting from a prolonged hospital stay or intubation, such as deep venous thrombosis and pneumonia.^24^, ^25^ Second, pre‐operative malnutrition is often seen in frail patients with advanced heart failure and has been associated with worse clinical outcomes, including mortality after LVAD implantation.^26^ Thus, pre‐LVAD implant malnutrition may partially explain these adverse events.^27^ In addition, the pro‐inflammatory environment seen in frailty can promote arrhythmogenesis and lead to sudden death by increasing oxidative stress via releasing reactive oxygen species.^28^

Albeit not statistically significant, frail pre‐operative patients had longer post‐procedure LOS and time‐to‐extubation after LVAD implantation in our study. Frailty has been previously significantly associated with longer intubation times after LVAD surgery.^29^, ^30^ Few studies have also reported increased LOS during index hospitalization for LVAD implantation.^23^, ^24^ The lack of statistical significance of the above findings may be due to our small sample size. However, these findings signify that frail patients who experience longer recovery times are at a greater risk of adverse outcomes from a prolonged hospital stay.

We hypothesized that 30‐day re‐admission rates would be greater among frail patients compared with non‐frail patients. However, 30‐day re‐admission was not statistically significant between the CFS‐defined non‐frail and frail cohort after LVAD implantation (35% vs 46%, *P* = 0.419). It is possible that the small sample size may have reduced the statistical power to detect the differences in the readmission rates. In a previous study, statistical significance was observed between the frail and non‐frail groups, although the outcome was reported as a composite of all‐cause mortality and 30‐day re‐admission.^31^ Although limited by the small sample size, another study reported opposing results.^32^ The investigators used multiple frailty parameters or surrogates and reported that frailty status according to hand grip strength were less likely to be re‐admitted within 30 days of discharge after LVAD implantation.^32^

Notwithstanding, 30‐day re‐admissions are common after LVAD implantation and they are associated with increased all‐cause mortality.^33^ Although some studies have reported rehospitalization over their whole study, data on the impact of frailty status on early re‐admission within 30 days are limited.

Therefore, more extensive multicenter studies are needed to study the influence of frailty on 30‐day rehospitalization after LVAD implantation.

Because there is no consensus on the optimal tool to assess frailty among patients with end‐stage heart failure undergoing LVAD implantation, various frailty assessment instruments have been previously utilized, each with its shortcomings.^4^ Few studies have also used substitutes for frailty, or the components of the phenotypic frailty model, such as cachexia,^34^ handgrip strength,^19^, ^26^ and sarcopenia.^4^, ^35^, ^36^

CFS as a frailty assessment tool may also be suitable for recognizing frail patients requiring further complete geriatric evaluation.^37^ CFS can easily be administered in a real‐life clinical setting and has a high correlation index with the frailty index.^16^ The CFS is a semiquantitative frailty tool that may be used by any health care professional without extra tests to assess frailty.^38^ CFS is also not subject to declines during hospitalization because it depends on function before arrival at the hospital.^37^ CFS‐defined frailty has been used in other cardiac populations to predict mortality, for instance, in patients with acute myocardial infarction,^37^ transcutaneous aortic valve replacement (TAVR),^39^, ^40^ percutaneous coronary interventions,^41^ and cardiac surgeries.^42^ According to our knowledge, this is the first study to use CFS in patients with advanced heart failure undergoing placement of LVAD. In our population of older patients ≥ 60 years that underwent LVAD, frailty was present in 40% pre‐operatively.

### 4.1 Limitations

There are some limitations to be considered when interpreting our findings. First, our study is a single‐center study and relatively small sample size. Thus, our results have the inherent problem of generalizability. The next step is corroborating these findings in a multicenter large‐scale sample of older patients. Although CFS has been validated for retrospective use,^17^ a limitation of our study is that CFS scores were determined retrospectively from existing medical records. However, CFS scores were assigned by geriatric medicine fellows well versed in frailty assessment. We followed the approach in prior studies.^17^, ^43^ We suggest that further studies comparing CFS to other methods of frailty assessment, such as Fried's frailty phenotype or Deficit Index, be conducted to determine which frailty tool best predicts outcomes after LVAD placement.

## 5 CONCLUSIONS

CFS‐defined frailty status was associated with 1‐year mortality after LVAD surgery and therefore CFS showed prognostic significance in 1 year survival. Further research should focus on reproducing our findings in a large‐scale study using prospective CFS assessment in older LVAD recipients.

## AUTHOR CONTRIBUTIONS

*Conceptualization, data analysis, data collection and writing of original draft:* Ajibawo. *Data collection, review and editing of the manuscript:* Chauhan. *Supervision and review of the manuscript:* Gopalan. *Conceptualization, supervision, review, and editing of the manuscript for intellectual content:* Agarwal. All the authors have read and agreed to the published version of the manuscript.

## FUNDING INFORMATION

This research did not receive any external funding.

## CONFLICT OF INTEREST

The authors declare no conflicts of interest regarding this study.

## ETHICS STATEMENT

This study was conducted in compliance with the ethical standards of our institution on human subjects as well as with the Helsinki Declaration (2103588228).
